# Milk Fatty Acids Composition Changes According to β-Hydroxybutyrate Concentrations in Ewes during Early Lactation

**DOI:** 10.3390/ani11051371

**Published:** 2021-05-12

**Authors:** Enrico Fiore, Anastasia Lisuzzo, Rossella Tessari, Nicoletta Spissu, Livia Moscati, Massimo Morgante, Matteo Gianesella, Tamara Badon, Elisa Mazzotta, Michele Berlanda, Barbara Contiero, Filippo Fiore

**Affiliations:** 1 Department of Animal Medicine, Production and Health, University of Padova, Viale dell’ Università 16, 35020 Legnaro, Italy; anastasia.lisuzzo@phd.unipd.it (A.L.); rossella.tessari@unipd.it (R.T.); massimo.morgante@unipd.it (M.M.); matteo.gianesella@unipd.it (M.G.); tamara.badon@unipd.it (T.B.); elisa.mazzotta@unipd.it (E.M.); michele.berlanda@unipd.it (M.B.); barbara.contiero@unipd.it (B.C.); 2 Department of Veterinary Medicine, University of Sassari, Via Vienna 2, 07100 Sassari, Italy; nicolettaspissu@gmail.com (N.S.); ffiore@uniss.it (F.F.); 3 Experimental Zooprophylactic Institute of Umbria and Marche, Via G. Salvemini, 06126 Perugia, Italy; l.moscati@izsum.it

**Keywords:** β-Hydroxybutyrate, ewes, gas-chromatography, milk fatty acids

## Abstract

**Simple Summary:**

Ketosis can occur during the last six weeks of gestation and continue to the early weeks of lactation due to an increase in energy requirement. This condition can cause substantial economic issues because of the decrease in production, the cost of medical management, the loss of the mothers and the lambs. A better knowledge of this disorder and its early diagnosis could make treatment more effective and optimize productivity. The aims of this study were to understand the metabolic status of the early-lactating ewes and to identify biomarkers for precocious diagnosis of subclinical ketosis using gas chromatographic technique. Different relationships were found between milk fatty acids and metabolic status of the ewes. Furthermore, 8 potential biomarkers were determined.

**Abstract:**

Ketosis is a metabolic disease of pregnant and lactating ewes linked to a negative energy balance which can cause different economic losses. The aims of this study were to understand the metabolic status of the early-lactating ewes and to identify biomarkers for early diagnosis of subclinical ketosis. Forty-six Sarda ewes were selected in the immediate post-partum for the collection of the biological samples. A blood sample from the jugular vein was used to determine β-Hydroxybutyrate (BHB) concentrations. Animals were divided into two groups: BHB 0 or healthy group (*n* = 28) with BHB concentration < 0.86 mmol/L; and BHB 1 or subclinical ketosis (*n* = 18) with a BHB concentration ≥ 0.86 mmol/L. Ten mL of pool milk were collected at the morning milking for the analyses. The concentration of 34 milk fatty acids was evaluated using gas chromatography. Two biochemical parameters and 11 milk fatty acids of the total lipid fraction presented a *p*-value ≤ 0.05. The study revealed different relationships with tricarboxylic acid cycle, blood flows, immune and nervous systems, cell functions, inflammatory response, and oxidative stress status. Eight parameters were significant for the receiver operating characteristic (ROC) analysis with an area under the curve greater than 0.70.

## 1. Introduction

Ketosis is a metabolic disorder associated with a negative energy balance (NEB), that can develop in the ewes from the last six weeks of gestation (pregnancy toxemia; OPT) and continue to the early weeks of lactation (lactational ketosis) [1,2]. Energy requirements for fetal growth increase of 150% in single-bearing ewes and of 200% in twins pregnancy [3], consequently the twins pregnancy have higher risk to develop ketosis. For this reason, OPT is often defined as twin-lamb disease and affects ewes with a parity of two or more new-born [3,4].

The main cause of ketosis is an alteration of energetic metabolism, with a reduction of glycemia and increase in synthesis of non-esterified fatty acids (NEFA) from adipose tissue lipolysis [5]. NEFAs can be (*a*) completely oxidized in tricarboxylic acid cycle, (*b*) converted in ketone bodies (acetoacetate, β-hydroxybutyrate (BHB) and acetone) or (*c*) esterified in triacyl-glycerol (TAG) in hepatic tissue [6,7,8]. The increase in BHB level is also a common finding in field. For this reason, BHB concentration is considered related to this metabolic disease in ruminants [9,10].

The BHB cut-off for subclinical ketosis is above 0.86 mmol/L, whereas BHB concentration above 3.0 mmol/L associated with clinical signs is indicative of clinical ketosis [9,11]. The characteristic signs of ketosis are anorexia, muscular weakness, depression and prolonged recumbency [3]. This metabolic disorder is characterized by hyperketonemia and hypoglycemia [5]. The low glycemia can lead to low glucose concentration in nervous system resulting in neurologic signs as, for example, cecity and ataxia [11]. A peculiarity of ketosis is the gradual decrease in milk production, associated with an increase in milk fat percentage and a decrease in milk protein percentage [12]. Milk contains more than 400 fatty acids of which 95% are triglycerides [13]. They can derive from (*a*) diet and rumen (biohydrogenation, bacterial degradation and synthesis) for the 40–45%, (*b*) de novo synthesis in the mammary gland for the 50% and (*c*) body fat mobilization for less than 10% [13,14].

The main economic losses due to ketosis are decrease in milk production, the cost of medical management, or the culling of the mothers and/or of the lambs [1]. In fact, the impaired metabolism influences biological substances availability for lamb growth, with an increased risk of low birth weight, perinatal mortality and reduction of lambs’ performance [15]. A better knowledge of this disease would significantly improve the productivity [15] and the early diagnosis would allow a specific and effective management and medical treatment [4].

On the basis of these assumptions, we aimed to deepen our knowledge of the metabolic status of ewes in early lactation and to identify biomarkers for early diagnosis of subclinical ketosis using gas chromatographic technique (GC).

## 2. Materials and Methods

### 2.1. Animals

In this study we enrolled a flock of high-yielding Sarda dairy sheep located in a commercial farm in North Sardinia (Italy) consisted in 800 lactating ewes housed in free stall barns and milked twice a day. Average daily milk yield from lambing to 30 DIM was 1.234 ± 0.34 kg/day and the length of lactation was 245 ± 24 days in this study. All ewes were fed a total mixed ration (TMR) composed of 700 g of haylage banded, 400 g of hay, 200 g of silage maize, 150 g of soya, 150 g of flaked corn and 150 g of beet pulp according to animals’ physiological and productive status. Milking sheep were fed a TMR formulated for lactating sheep (40–50 kg of body weight—BW) with an average milk production of 1.1 kg/day for a standard lactation. The protein content was 15% DM and the energy value of the ration was 9.5 ME (Mj/kg DM). The ewes grazed 1 h/day natural pasture.

The experiment was conducted in 46 adult ewes: 11 primiparous, 11 animals with 2 parities, 10 with 3 parities, and 14 with ≥ 4 parities. All animals lambed twins and were evaluated at 7 ± 3 days in milk (DIM), considering that ketosis is reported most frequently in the period between the sixth week before lambing and the first weeks of lactation [5,16,17]. In each enrolled animal, the body condition score (BCS) was evaluated on a scale of 1 to 5 points before clinical examination and blood sampling [18]. Age and parity were also considered as characteristics of ewes. Healthy status of animals was evaluated by veterinarians of University of Sassari (Italy) and were clinically healthy.

### 2.2. Experimental Design

Blood samples were collected at 7 ± 3 DIM from the jugular vein through vacutainer system and stored in tubes containing a clot activator (9 mL; Terumo Venosafe, Leuvel, Belgium). The concentration of BHB was measured before blood collection using a portable digital reader (Abbott Precision Xtra™ meter, Oxon, UK) and blood ketone test strips (Abbott Precision Xtra™ Blood Ketone test strips, Oxon, UK). Milk samples were collected at the morning milking. Ewes enrolled in the study did not show signs of mastitis during lactation and the somatic cell count (SCC) was ≤ 500.000 cell/mL, index of good health of the mammary gland [19]. After washing, drying, pre-dipping and fore stripping, we have collected manually 5 mL of milk from each emi-mammary gland of each ewe. The biological material was refrigerated at 4 °C until transported to the laboratory of the University of Sassari (Italy) within 1 h from the sampling.

The blood samples containing clot activator were centrifuged at 3000 rpm × 10 min in the laboratory (Hettich^®^ EBA 20 centrifuge, Stuttgart, DE, Germany). The serum was extracted and stored at −20 °C until biochemical analysis. The two milk samples of each ewe were stirred in order to obtain a pool milk of 10 mL and it was stored at −80 °C until the GC analysis. Two aliquots of serum and one aliquot of pool milk from each ewe were sent to University of Padua (Italy), at Department of Animal Medicine, Production and Health (MAPS) in dry ice packaging within 24 h for the analysis.

### 2.3. Blood Analysis

Serum biochemistry analyses was performed by the laboratory of the Experimental Zooprophylactic Institute of Umbria and Marche (IZSUM, Perugia, Italy). The serum was assessed employing automatic clinical chemistry analyzer (Konelab 200, Cornaredo, Italy). Serum BHB concentrations were measured using β-hydroxybutyrate Enzymatic Kinetics (Randox, Crumlin, UK; BHB, mmol/L). Serum NEFA concentration was determined with the NEFA RX Monza test colorimetric method (Randox, Crumlin, UK). Glucose was measured using colorimetric test (Sclavo Diagnostic Dasit-Italy, Cornaredo, Italy) and urea using a kinetic method (Sclavo Diagnostic Dasit-Italy, Cornaredo, Italy).

The ewes were divided into two groups depending on their BHB blood concentration. BHB 0 group or healthy group enrolled 28 animals with blood value of BHB below 0.86 mmol/L [9,11]. The BHB 1 group, or sick group, enrolled 18 animals with blood value of BHB above cut-off, considered hyperketonemic group.

### 2.4. Milk Analysis

Milk gas chromatography (GC) was performed by the laboratory of MAPS. The samples were exposed to two different procedures sequentially: directly methylation by HCl-methanol of the carbon chain to perform GC analysis, and mixing every milk sample with internal standards (C9 and C15 triacylglycerols) to perform quantification of fatty acids.

The 3N methanolic hydrochloric acid was used for the methylation process of the milk lipids. The samples obtained were placed in an oven for one hour at 100 °C and then neutralized with a solution of potassium carbonate (K_2_CO_3_). Finally, 34 fatty acids were obtained for each milk sample. The fatty acid methyl esters were separated and quantified in split less mode by GC using a TRACE GC/MS (Thermo Quest, Milan, Italy) equipped with a flame ionization detector (FID) and a polar fused-silica capillary column (Capillary Column Omegawax, 30 m× 0.25 mm× 0.2 µm film). Helium was used as the carrier gas at a flow rate of 1 mL/min. Data for plasma fatty acid were calculated in mg/dL.

### 2.5. Statistical Analysis

Biochemical parameters, BCS, DIM, parity and milk fatty acids data were analyzed using the SAS system software (version 9.4; SAS Institute Inc., Cary, North Carolina, USA). A one-way ANOVA was used to evaluate the differences of the total lipid fraction within the two groups (BHB0 vs. BHB1). This test was used to select the parameters with a predictive power in order to diagnose subclinical ketosis or hyperketonemia. The hypotheses of linear model on the residuals were graphically assessed.

The Receiver Operating Characteristic (ROC) (MedCalc Sofware Ltd., Ostend, Belgium) curves were performed to establish threshold value of each predictive biochemical parameter or forecast milk fatty acids. The ROC curves were derived from the analysis of data of all experimental animals (BHB0-BHB1) with the discriminant of ewes with BHB > 0.86 mmol/L (BHB1) [9,11]. The area under the curve (AUC) shows the diagnostic power of the test. Therefore, the optimal cut-off of each parameter based on Youden criterion, was calculated to discriminate the subclinical ketosis or hyperketonemia groups in ewes in early phase of lactation. Statistical significance was set at *p*-value ≤ 0.05.

## 3. Results

Table 1 shows the mean values ± standard error of the mean (SEM) regarding BHB, NEFA, glucose, urea, DIM, body condition score (BCS) and parity for all enrolled animals, based on the two identified BHB groups (BHB0 and BHB1). A statistically significant difference between BHB0 and BHB1, was found for BHB (*p* < 0.001), glucose (*p* = 0.009) and urea (*p* = 0.007) (Table 1). Parity, BCS, DIM and NEFA presented no significant difference (*p* > 0.05).

The mean value of the different fatty acids of milk lipid fraction was compared to change in blood concentration of BHB of the two different groups of ewes (Table 2).

In the milk samples were identified 34 different fatty acids (Table 2), of which 3 medium chain fatty acids (MCFA) (C6:0, C8:0 and C10:0); 12 long chain fatty acids (LCFA) (C14:0, C14:1 ω 5, C16:0, C16:1 ω 7, C16 DMA, C18, C18:1 ω 9, C18:1 ω 7, C18:2 ω 6, C18:3 ω 6, C18:3 ω 3 and C18:4 ω 3) and 19 very long chain fatty acids (VLCFA) (C20:0, C20:1 ω 9, C20:1 ω 7, C20:2 ω 6, C20:3 ω 9, C20:3 ω 6, C20:4 ω 6, C20:3 ω 3, C20:4 ω 3, C20:5 ω 3, C22:0, C22:1 ω 9, C22:2 ω 6, C22:4 ω 6, C22:5 ω 6, C22:5 ω 3, C22:6 ω 3, C24:0 and C24:1 ω 9).

Among identified fatty acids, 11 had significant difference between BHB0 and BHB1, specifically 5 long chain fatty acids [C18 (*p* = 0.0002), C18:1 ω 9 (*p* = 0.042), C18:3 ω 6 (*p* = 0.001), C18:3 ω 3 (*p* = 0.038), C18:4 ω 3 (*p* = 0.023)] and 6 very long chain fatty acids [C20 (*p* = 0.002), C20:3 ω 9 (*p* = 0.013), C20:3 ω3 (*p* = 0.049), C20:5 ω 3 (*p* = 0.015), C24 (*p* = 0.047) and C24:1 ω 9 (*p* = 0.050)].

The analysis of ROC curves was performed on the significant milk fatty acids and significant biochemical parameters (Table 3). The ROC curve established the threshold of each parameter for the diagnosis of subclinical ketosis or Hyperketonemia (HK) (BHB > 0.86 mmol/L). The reliability of the cut-off as a biomarker of metabolic diseases depends on the area under the curve (AUC): if the AUC is greater than 0.70 the test performed for the biochemical parameters and milk fatty acids is considered predictive. For values of AUC ≥ 0.8 are diagnostic test is considered good, and for values ≥ 0.90 the accuracy is considered excellent. We selected glucose (AUC = 0.72) and urea (AUC = 0.73) as biochemical parameters with AUC ≥ 0.70.

**Figure 1:**
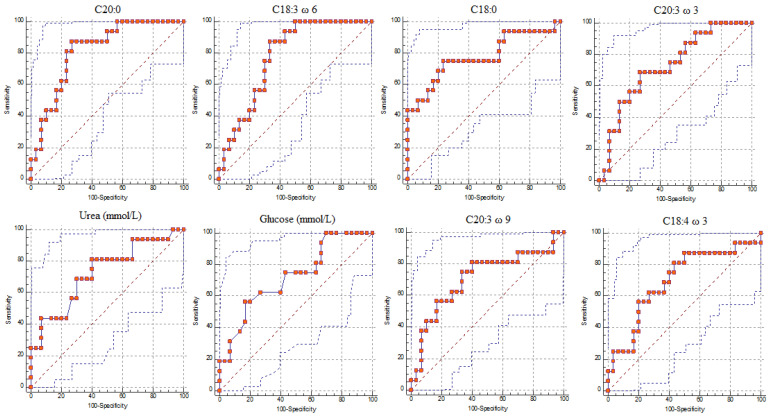
Receiver Operating Characteristic (ROC) curves of biochemical parameters and fatty acids having Area Under the Curve (AUC) greater than 0.70 listed in decreasing predictive function.

In Figure 1 were reported the ROC curves of the biochemical parameters and of milk fatty acids with AUC greater of 0.70.

## 4. Discussion

The present study described the metabolic status of early-lactating ewes and analyzed potential biomarkers for the precocious diagnosis of subclinical ketosis.

Ketosis can affect adult ewes during late gestation, early and late lactation. This disorder can influence dams and lambs’ health, productivity and enhance economic losses [1]. Therefore, an early diagnosis is essential for an effective treatment [4]. Furthermore, animal health influences both the performance and the milk’s quality. In fact, milk composition is related to the metabolic status with profound changes during lactation [13].

The aims of this study were to evaluate milk fatty acids composition of healthy and hyperketonemic ewes using GC to describe the metabolic status of animals in early lactation and to identify potential biomarkers for the diagnosis of the onset of ketosis.

Among blood parameters analyzed, only BHB, glucose and urea showed a significant change between the two groups (BHB0-BHB1). The mean values of BHB and urea were greater in sick animals (1.35 mmol/L and 7.66 mmol/L, respectively) compared to healthy animals (0.63 mmol/L and 6.08 mmol/L, respectively. Besides, the mean values of glucose were greater in BHB0 group (4.07 mmol/L) than in BHB1 group (3.43 mmol/L). Hyperketonemia, hypoglycemia and uremia were reported in BHB1 group: these findings are consistent with an early ketosis due to the increase in protein and fat metabolism [20].

NEFAs derive from body fat mobilization and they increase during ketosis [21]. NEFAs concentrations are positively related to milk’s fat synthesis, so they represent the crucial biological substances for it [12]. However, both NEFAs concentration and total milk fatty acids were not significantly increased in BHB1 or hyperketonemic group.

The long chain fatty acids (LCFA) are mobilized from adipose tissue during a negative energy balance (NEB) status. Among LCFA, C16:0 (palmitic acid), C18:0 (stearic acid) and C18:1 (oleic acid) are the main fatty acids mobilized in NEB condition [8]. According to the literature, Zhang et al. (2013) [22] reported that in dairy cows the plasmatic concentrations of C16:0 and C18:0 increase during ketosis. C16:0 is reported as the LCFA most present in milk fat [14]. Moreover, C16:0 derives also from de novo synthesis in mammary gland, which produces fatty acids with maximum 16 carbons [13]. C16:0 increase in milk fat more than C18:0 in accordance to severity of NEB [14].

In our study, C16:0 was not different between the two groups, but C18:0 increased in hyperketonemic group compared to healthy group. This may suggest a mild NEB condition in BHB1 group despite the high BHB concentrations reported. The increase of NEFAs, particularly C18:0, promote the expression of cell death-inducing DFFA like effector a (CIDEA) protein. This protein is related to milk fat synthesis and secretion in mammary epithelial cells [12]. C18:0 also promotes triglycerides synthesis and gene expression. It increases the sterol regulatory element binding protein (SREBP-1) genes and consequently the expression of the enzyme stearoyl-CoA desaturase 1 (SCD1). This enzyme is involved in mono-unsaturated fatty acids (MUFAs) synthesis, with an influence on milk fatty acid composition [12,14]. In our study, the significant MUFAs were C18:1 ω 9 (oleic acid) and C24:1 ω 9 (nervonic acid) which were raised in BHB1 group. Consequently, the increment of C18:0 was related to MUFAs’ increment due to the activation of SCD1 [12,14].

C18:0 influences also the pyruvate carboxylase promoters to stimulate gluconeogenesis and maintain oxaloacetate for the tricarboxylic acid cycle [14]. This cycle is highly important in mammary epithelial cells for ATP and NADPH production in fatty acid synthesis [23]. Furthermore, alterations in cholesterol synthesis and concentration during ketosis [8,23] could affect mitochondria associated endoplasmic reticulum membrane (MAM), a calcium carrier into mitochondria. The higher concentration of unsaturated fatty acids (UFA) and the lower concentration of saturated fatty acids (SFA) indicate a reduction of de novo fatty acid synthesis dependent from mitochondria activity, where the tricarboxylic acid cycle takes place [23,24].

In our study we identified 9 SFA and 24 UFA. Among SFA, 3 fatty acids (C18:0 (*p*-value = 0.0002), C20:0 (*p*-value = 0.002), and C24:0 0 (*p*-value = 0.047)) significantly increased in BHB1 group. Among UFA, 8 fatty acids C18:1 ω 9 (*p*-value = 0.042), C18:3 ω 6 (*p*-value = 0.001), C18:3 ω 3 (*p*-value = 0.038), C18:4 ω 3 (*p*-value = 0.023), C20:3 ω 9 (*p*-value = 0.013), C20:3 ω 3 (*p*-value = 0.049), C20:5 ω 3 (*p*-value = 0.015), and C24:1 ω 9 (*p*-value = 0.050)) significantly increased in BHB1. These findings would suggest a possible early alteration in tricarboxylic acid cycle in BHB1 group, particularly targeting de novo synthesis of fatty acids in mammary gland.

According to Loften et al. (2014) [14], the blood concentrations of C16:0 and C18:1 ω 9 increase after parturition, while C18:0 decreases. These results suggest that C18:0 in NEB condition may be involved in energy production through β-oxidation in liver and muscle, besides may be secreted in milk as C18:0 or C18:1 ω 9. In fact, 50% of C18:1 ω 9 in milk fat derives from desaturation of C18:0 [14]. Gross et al. (2019) [13] reported that the increase in C18:1 ω 9 concentrations in milk fat is detectable before BHB, so C18:1 ω 9 may be considered as a predictive factor for subclinical ketosis. According to these previous results, our study reported that C18:1 ω 9 was significantly increased in hyperketonemic group (BHB1) compared to healthy group (BHB0), suggesting the increase in C18:1 ω 9 as an early biomarker for subclinical ketosis in ewes.

In our study, α-linolenic acid (C18:3 ω 3; ALA) and γ -linoleic acid (C18:3 ω 6; GLA) were significantly increased in hyperketonemic group. ALA is a polyunsaturated omega-3 fatty acid (PUFA ω-3) while GLA is a polyunsaturated omega-6 fatty acid (PUFA ω-6) [25]. Both PUFA ω-3 and PUFA ω-6 are activators of peroxisome proliferator-activated receptors (PPARs) α, β, γ and substrate for lipid mediators such as eicosanoids. However, C18:3 ω 6 acid enhances the production of prostaglandins, thromboxanes and lipoxins with anti and pro-inflammatory effects, while C18:3 ω 3 acid enhances the production of lipoxins, resolvins and protectins with anti-inflammatory effects [25]. The C18:3 ω 3 is one of the most prominent PUFA in milk fat [26,27]. It is an inhibitor of nuclear factor κB (NFκB) such as others PUFA ω-3, promoting anti-inflammatory effects due to the limiting production of inflammatory cytokines (IL-1 β, IL-6, TNFα) and the down regulation of cyclo-oxigenase-2 protein (COX-2) expression. Conversely, the increment of SFA (C18:0, C20:0 and C24:0) can activate NFκB and induce the transcription of several adhesion molecules such as intercellular adhesion molecules (ICAM-1), endothelial leukocyte adhesion molecules (ELAM-1) and vascular cell adhesion molecules (VCAM-1). They activate toll-like receptor 4 (TLR-4) that induce an increased production of nitric oxide that enhances blood flow to muscle [25]. In BHB1 group, these alterations in inflammatory response, resulting from the increase in PUFA ω 3 and PUFA ω 6, may promote the muscular proteins mobilization to supply the energy requirement in NEB.

The stearidonic acid (C18:4 ω 3) can derive from Δ6-desaturation of C18:3 ω 3 [26] using the metabolic pathway of α-linolenic acid metabolism. In our study, C18:4 ω 3 was statistically significant milk fatty acid increased in hyperketonemic group. It is a PUFA ω-3 that can be converted in eicosatetraenoic acid (C20:4 ω 3; ETA) through elongase enzyme and subsequently in eicosapentaenoic acid (C20:5 ω 3; EPA) through Δ5-desaturation [26,28]. The transfer efficiency of C18:4 ω 3 in milk fat is around 39%, although considering the enzymatic products (ETA and EPA) the efficiency increase up to 47% [26]. In our study, ETA was mainly increased in BHB1 group, even if it was not significant. Instead, EPA presented a significant increase in hyperketonemic group. EPA is an anti-inflammatory and anti-thrombotic PUFA ω-3. In fact, it is a precursor of prostaglandin-3 which inhibits platelets aggregation and can be used to produce protectins [28] and resolvins by COX-2, lipoxygenase or epoxygenases [29]. Furthermore, EPA influences immune system function due to (*a*) an inhibitory effect of cell surface expression of major histocompatibility complex II (MHC-II) to antigen presentation; (*b*) an increased IL-4 production from T-lymphocytes; (*c*) a reduction of prostaglandin E2 production which is a potent inhibitor of lymphocytes activity and proliferation [30]. Free form of EPAs are incorporated in cell membrane phospholipids and replace arachidonic acids [29]. The changes in cell membrane lipid profiles could influence cellular functions such as cellular membrane fluidity [31]. This context could suggest an alteration of inflammatory response, immune system and cells functions.

Cellular membrane fluidity is sustained by mead acid (C20:3 ω 9) to maintain cell viability and growth. This fatty acid derives by enzymatic elongation and desaturation of C18:1 ω 9 in response to a lack of C18:3 ω 3 and linoleic acid (C18:2 ω 6) [32]. In our study, milk C20:3 ω 9, C18:3 ω 3 and C18:2 ω 6 increased in hyperketonemic ewes, even if C18:2 ω 6 was not significant. The decrease of serum C18:3 ω 3 and C18:2 ω 6 concentrations in BHB1 group may be due to their use in inflammatory response and mammary excretion. As previously described, C20:3 ω 9 has critical properties for cellular membrane fluidity and it shows anti-inflammatory function: in fact, C20:3 ω 9 inhibits arachidonic transformation and can be converted into leukotrienes with a consequential decrement in inflammatory reactions and leukocytes infiltration [32,33,34].

Eicosatrienoic acid (C20:3 ω 3; ETE) is a PUFA ω-3 bounding PPARγ and NFκB with anti-inflammatory effects as previously mentioned [35]. Furthermore, ETE shows a higher antioxidant activity compared to C18:3 ω 3, similar to docosahexaenoic acid (C22:6 ω 3; DHA) and docosapentaenoic acid (C22:5 ω 3; DPA), and lower than EPA [36]. All these milk fatty acids increased in hyperketonemic ewes, particularly ETE, C18:3 ω 3 and EPA that were statistically different between groups. However, PUFAs mobilization increases lipid peroxidation and may stimulate the production of reactive O_2_ species (ROS) by the mitochondria, overcoming systemic antioxidant factors. This ROS production results in an oxidative stress and a reduction in immune system efficiency [37,38]. The mobilization of PUFA ω 3 and PUFA ω 6 seems to be closely associated with the regulatory mechanisms of the inflammatory response, immune functions and oxidative stress state.

Nervonic acid (C24:1 ω 9) can be synthetized by C18:1 ω 9: through the mammary secretion in milk, it represents an important integrative support for brain development. Higher concentration of serum C24:1 ω 9 has been associated with lower risk of Alzheimer’s disease, an autoimmune disease related to demyelination and metabolism of lipids in human patients, thus it is considered positively associated with nervous system health status [39]. In our study, C24:1 ω 9 was significantly increased in hyperketonemic group (BHB1): this result may be considered as a protective response in ketotic animals for preventing neurological damages, besides further investigations would be useful to better understand the actual relationship between C24:1 ω 9 mobilization and neurological syndrome.

Among biochemical parameters, glucose and urea were recognized as possible biomarkers, presenting an AUC equal to 0.72 and 0.73 respectively. Furthermore, the significant milk fatty acids were analyzed for a receiver operating curve (ROC) to identify potential biomarkers (Table 3). Five milk fatty acids (C18:1 ω 9, AUC = 0.67; C18:3 ω 3, AUC = 0.65; C20:5 ω 3, AUC = 0.68; C24, AUC = 0.68; C24:1ω9, AUC = 0.64) presented an AUC lower than 0.70, consequently they were not considered as predictive biomarkers. Six milk fatty acids (C20:0, AUC = 0.82; C18:3 ω 6, AUC = 0.78; C18:0, AUC = 0.77; C20:3 ω 3, AUC = 0.73; C20:3 ω 9, AUC = 0.71; C18:4 ω 3, AUC = 0.70) presented an AUC higher than 0.70, thus they were identified as accurate and potential biomarkers. These milk fatty acids showed a significant increase in hyperketonemic animals establishing different cut-offs with a confidence interval of 95% (9.65 mg/dL; 7.07 mg/dL; 465.31 mg/dL; 0.45 mg/dL; 2.07 mg/dL and 3.37 mg/dL, respectively).

## 5. Conclusions

In our study, the trends of milk fatty acids related to blood concentrations of BHB were described and 6 early subclinical ketosis predictive fatty acids and 2 biochemical parameters were identified. The change in milk fatty acids composition during the first days of lactation confirmed the applicability of GC as a predictive technique for the diagnosis of hyperketonemia.

The identification of significant fatty acids as early biomarkers for subclinical ketosis showed the close relationships between different inflammatory pathways and systemic impaired function in NEB condition as (1) changes in milk fatty acids composition, (2) tricarboxylic acid cycle, (3) blood flows, immune and nervous systems, (4) cell functions, inflammatory response, and oxidative stress status.

Future studies would be necessary to investigate the variations in the four-milk lipid class according to BHB serum concentration and to clarify the relationship with lipids mobilization, biological functions and metabolic status, in order to improve diagnosis and treatment of ewes with hyperketonemia.

## Figures and Tables

**Table 1:** Mean value with standard error of the mean (±SEM) of ewes’ data and biochemical parameters in the two groups (BHB0 and BHB1).

Parameters	BHB 0 (*n* = 28)	SEM	BHB 1 (*n* = 18)	SEM	*p*-Value
BHB (mmol/L) ^1^	0.63	0.12	1.35	0.35	<0.001
NEFA (mmol/L) ^2^	0.17	0.04	0.27	0.05	NS ^3^
Glucose (mmol/L)	4.07	0.14	3.43	0.19	0.009
Urea (mmol/L)	6.08	0.33	7.66	0.45	0.007
DIM ^4^	4.88	0.66	4.08	1.22	NS ^3^
BCS ^5^	3.13	0.69	2.61	1.13	NS ^3^
Parity	3.19	1.47	2.33	1.50	NS ^3^
Daily milk yield (kg/day)	1.25	0.06	1.22	0.04	NS ^3^

^1^ β-hydroxybutyrate, ^2^ Non-Esterified Fatty Acid, ^3^ not significant, ^4^ days in milk, ^5^ body condition score.

**Table 2:** Milk fatty acids mean values with standard error of the mean (±SEM) related to the Total Lipid Class in the two groups (BHB0-BHB1).

Fatty Acids	Name	BHB 0 (*n* = 28)	SEM	BHB 1 (*n* = 18)	SEM	*p*-Value
C6	Caproic acid	154.91	55.28	285.32	75.69	NS ^1^
C8	Caprylic acid	150.01	15.10	141.04	20.68	NS ^1^
C10	Capric acid	390.30	37.51	404.01	51.37	NS ^1^
C14	Myristic acid	418.09	22.52	383.35	30.84	NS ^1^
C14:1 ω 5	Myristelaidic acid	10.58	1.51	14.28	2.07	NS ^1^
C16	Palmitic acid	1163.05	62.34	1137.56	85.36	NS ^1^
C16:1 ω 7	Palmitoleic acid	42.95	5.50	51.07	7.53	NS ^1^
C18	Stearic acid	396.36	25.45	570.88	34.85	0.0002
C18:1 ω 9	Oleic acid	1008.32	115.75	1418.64	158.50	0.042
C18:1 ω 7	Cis-Vaccenic acid	77.45	12.68	98.40	17.36	NS ^1^
C18:2 ω 6	Linoleic acid	175.99	7.90	189.38	10.82	NS ^1^
C18:3 ω 6	γ -linolenic acid	6.52	0.26	8.03	0.35	0.001
C18:3 ω 3	α-linolenic acid	22.87	1.34	27.73	1.83	0.038
C18:4 ω 3	Stearidonic acid	3.87	0.48	5.80	0.66	0.023
C20	Arachidic acid	9.19	0.64	12.71	0.87	0.002
C20:1 ω 9	Gondoic acid	9.96	1.32	14.41	1.81	0.053
C20:1 ω 7	Paullinic acid	5.79	0.54	7.53	0.75	NS ^1^
C20:2 ω 6	Eicosadienoic acid	2.14	0.16	2.14	0.22	NS ^1^
C20:3 ω 9	Mead acid	1.92	0.09	2.33	0.13	0.013
C20:3 ω 6	Dihomo- γ -linolenic acid	1.76	0.10	1.77	0.14	NS ^1^
C20:4 ω 6	Arachidonic acid	18.49	0.94	18.32	1.29	NS ^1^
C20:3 ω 3	Eicosatrienoic acid (ETE)	0.43	0.03	0.55	0.05	0.049
C20:4 ω 3	Eicosatetraenoic acid (ETA)	0.18	0.02	0.22	0.03	NS ^1^
C20:5 ω 3	Eicosapentaenoic acid (EPA)	18.22	1.31	23.87	1.79	0.015
C22	Behenic acid	0.37	0.12	0.62	0.16	NS ^1^
C22:1 ω 9	Erucic acid	0.48	0.10	0.61	0.14	NS ^1^
C22:2 ω 6	Docosadienoic acid	0.19	0.01	0.18	0.02	NS ^1^
C22:4 ω 6	Adrenic acid	3.30	0.18	3.30	0.24	NS ^1^
C22:5 ω 6	Docopentaenoic acid	0.02	0.01	0.01	0.01	NS ^1^
C22:5 ω 3	Decosapentaenoic acid (DPA)	7.54	0.58	8.42	0.80	NS ^1^
C22:6 ω 3	Docosahexaenoic acid (DHA)	3.28	0.37	4.21	0.51	NS ^1^
C24	Lignoceric acid	2.15	0.15	2.68	0.21	0.047
C24:1 ω 9	Nervonic acid	1.20	0.07	1.43	0.09	0.050
C16 DMA	Dimethyl-acetal-palmitic acid	14.45	4.46	27.30	6.11	NS ^1^
Mg FA/dl		4536.63	245.66	5276.94	336.38	NS ^1^

NS ^1^: Not significant.

**Table 3:** Results related to the ROC curves of the predictive parameters for the development of subclinical ketosis (BHB1).

Parameters	Cut-off (mg/dl)	AUC ^1^	Se ^2^	95% CI for Se	Sp ^3^	95% CI for Sp	+LR	*p*-Value
C20	>9.65	0.82	87.5	61.7–98.4	73.33	54.1–87.7	3.28	<0.001
C18: 3 ω 6	>7.07	0.78	87.5	61.7–98.4	66.67	47.2–82.7	2.62	<0.001
C18	>465.31	0.77	75	47.6–92.7	76.67	57.7–90.1	3.21	<0.001
C20: 3 ω 3	>0.45	0.73	68.75	41.3–89.0	73.33	54.1–87.7	2.58	<0.001
C20: 3 ω 9	>2.07	0.71	75	47.6–92.7	66.67	47.2–82.7	2.25	<0.001
C18: 4 ω 3	>3.37	0.70	81.25	54.4–96.0	56.67	37.4–74.5	1.87	<0.001
C18: 1 ω 9	>1277.52	0.67	56.25	29.9–80.2	83.33	65.3–94.4	3.38	<0.001
C20: 5 ω 3	>22.33	0.68	56.25	29.9–80.2	80	61.4–92.3	2.81	<0.001
C24	>2.03	0.68	81.25	54.4–96.0	53.33	34.3–71.7	1.74	<0.001
C18: 3 ω 3	>21.62	0.65	87.5	61.7–98.4	43.33	25.5–62.6	1.54	<0.001
C24:1 ω 9	>0.96	0.64	93.75	69.8–99.8	33.33	17.3–52.8	1.41	<0.001

^1^ Area Under the Curve; ^2^ Sensitivity; ^3^ Specificity.

## Data Availability

The data will be available by sending an email to the corresponding author.
